# Surviving on Limited Resources: Effects of Caloric Restriction on Growth, Gene Expression and Gut Microbiota in a Species With Male Pregnancy (*Hippocampus erectus*)

**DOI:** 10.1111/mec.17754

**Published:** 2025-04-07

**Authors:** Freya Adele Pappert, Vincent Alexander Wüst, Carmen Fontanes Eguiguren, Olivia Roth

**Affiliations:** ^1^ Marine Evolutionary Biology, Zoological Institute Christian‐Albrechts‐Universität Kiel Kiel Germany; ^2^ Evolutionary Ecology of Marine Fishes Helmholtz‐Centre for Ocean Research Kiel (GEOMAR) Kiel Germany; ^3^ University of Vienna Vienna Austria

**Keywords:** caloric restriction, life‐history, male pregnancy, resource allocation, seahorse

## Abstract

Caloric restriction (CR) studies have traditionally focused on species with conventional reproductive roles, emphasising female's greater investment in costly gametes and parental care. While the divergent impact of CR on males and females is evident across species, the factors driving this variation, that is, resource allocation to reproductive elements as part of distinct life history strategies, remain unclear. To address this, we investigated the effects of CR on development, gene expression and intestinal microbiota in the lined seahorse *Hippocampus erectus,* a species with male pregnancy, where fathers invest in offspring through gestation. Juvenile seahorses were subjected to ad libitum (AL) or CR feeding for 5 months. CR stunted male growth and brood pouch development, reflecting the energy demands of this crucial parental care trait. However, condition index declined in CR females but not males, while ovarian weight remained unchanged. Transcriptome analysis demonstrated organ‐ and sex‐specific responses to CR with distinct lipid and energy‐related pathways activated in male and female livers, indicative of survival enhancement strategies. CR had minimal impact on genes associated with spermatogenesis, but downregulated lipid metabolic and inflammatory genes in ovaries, emphasising the importance of pre‐copulatory resource allocation in female gametes. CR strongly shaped gut microbial composition, creating distinct communities from AL seahorses while also driving sex‐specific taxonomic differences. Our research indicates that nutrient limitation's impact on males and females is influenced by their allocation of resources to reproduction and parental investment. We underscore the significance of studying species with diverse reproductive strategies, sex roles and life‐history strategies.

## 1 Introduction

Females and males have distinct reproductive strategies, leading to specific energy allocation. Females typically invest more in reproduction than males, often through a combination of costlier games and greater parental care (Trivers 1972). For example, in many taxa, females produce energetically expensive eggs and bear additional burdens, such as pregnancy and lactation (Hayward and Gillooly 2011; Speakman 2008). As direct reproductive investment is often greater in females due to the combined costs of gamete production and parental care, males in many species allocate more energy to direct activities that enhance reproductive success, such as courting and mate guarding (Clutton‐Brock and Parker 1992; Janicke et al. 2016). These differences in reproductive strategies likely influence how males and females allocate resources and energy in response to environmental challenges, such as caloric restriction (CR).

Studies have shown that female mice and fruit flies tend to react stronger to CR in terms of improved health and lifespan compared to their male counterparts (Kane et al. 2018; Regan et al. 2016). In contrast, fasting extended the lifespan of male but not female killifish, potentially facilitated by sex‐specific expression changes (McKay et al. 2022). Despite these species‐specific sex differences, biomedical research has historically neglected sex differences in CR responses, underlining that the drivers of this sexual dimorphism are still poorly understood (Zucker et al. 2021; Pasin et al. 2023).

CR induces a state of hormetic stress, stimulating a scarcity of food and prompting it to conserve energy until more nutrients become available (Sinclair and Howitz 2005). According to Kirkwood's disposable soma theory, longevity requires investing in somatic maintenance, which takes priority over reproduction when resources are scarce (Kirkwood and Shanley 2005). By postponing reproduction during periods of limited energy availability, animals can extend their lifespan within the constraints of their maximum biological potential, resuming breeding when resources become abundant again (Masoro and Austad 1996). This strategy aims to maximise overall reproductive success (Shanley and Kirkwood 2006). Some species naturally have long periods of fasting during hibernations when food is scarce, including many reptiles and fish (Ikeya and Kume 2011; Wang et al. 2006). However, species with long lifespans, high caloric diets and high reproductive outputs, such as queen bees, ants and termites, challenge this theory (Keller 1998; Remolina and Hughes 2008). If done excessively, CR can have negative health impacts in humans (e.g., extreme weight loss, lethargy, diminished libido, etc.) (Shanley and Kirkwood 2006; Dirks and Leeuwenburgh 2006; Liao et al. 2010; Speakman and Mitchell 2011).

Despite the general consensus that different species showcase varying sexual dimorphism with respect to the effects of CR, most research to date has focused predominantly on species with conventional reproductive roles, with females showing increased investment into both gametes and offspring (Mitchell et al. 2015). However, sex roles in the animal kingdom are not constrained and have evolved with flexibility across species (Janicke et al. 2016; Benvenuto et al. 2017; Smith and Wootton 1995), leaving a gap in our understanding regarding whether the sex‐specific effects of fasting are primarily driven by differences in resource allocation to gamete production (e.g., egg vs. sperm) or by broader sexual reproductive roles, such as parental care. Understanding the ultimate drivers of sexual dimorphism in the effects of CR on health across different species holds significant implications for integrating concepts of sexual selection into medical sciences. This knowledge can offer valuable insights for CR interventions in sex‐ and gender‐specific health or the development of CR mimetics, paving the way for improved strategies to enhance well‐being and longevity (Balasubramanian et al. 2017).

To bridge the existing research gap, we designed an experiment focusing on how CR, implemented through intermittent fasting (IF), affects development, gut microbiota and aging pathways in a species with male pregnancy, the lined seahorse *Hippocampus erectus* . In this species, the female transfers her eggs to the male's pouch, which he then fertilises with his sperm. The embryos are embedded in the fully enclosed pouch containing blood vessels supplying nutrients and oxygen, on top of providing a controlled environment (Stölting and Wilson 2007; Lin et al. 2012). The unique reproductive biology of this species allows us to disentangle the cost of investment into the brooding structure (male) versus investment into egg production (female), which are two energetically costly traits typically found in females. This provides an opportunity to uncover how divergent life histories and reproductive trade‐offs affect sexual dimorphic responses to CR.

CR influences signalling pathways involved in growth, metabolism, oxidative stress response, damage repair, inflammation, autophagy and proteostasis, shaping the aging process and yielding positive physiological outcomes. The latter encompass improved cardiovascular health, reduced oxidative damage, enhanced DNA repair, promotion of autophagy and mitigating gut dysbiosis (Speakman and Mitchell 2011; López‐Otín et al. 2023; Mercken et al. 2012). Lifelong CR increases lifespan in many species, including yeast (Parrella and Longo 2008), nematodes (Braeckman et al. 2006), fruit flies (Burger et al. 2010) and rodents (Speakman and Mitchell 2011; Selman et al. 2008). Rejuvenating properties were suggested for mice, rhesus monkeys and humans fed on calorie‐restricted diets who showed DNA methylation patterns equal to their younger counterparts (Maegawa et al. 2017) and a younger, healthier microbial composition after 2 months of CR (Zeng et al. 2019).

We took 52 three‐month‐old juvenile (not sexually mature) seahorses, which were divided equally into two feeding groups: Ad libitum (AL) and calorie‐restricted (CR). Over the course of 5 months, the AL group was fed twice a day, while the CR group was fed every other day (also twice), resulting in a calorie restriction of approximately 50%. As seahorses lack a stomach, they are limited in the consumption of food at each food provisioning (Woods 2002). This avoids overeating in the AL group and ensures a restriction of calories in the fasting group. We measured the weight of the seahorses at four time points over the experimental period of 5 months. In males, we examined physiological developmental differences in pouch formation, the male critical reproductive structure. In females, we compared, at the end of the fasting period, the ovary masses to assess egg production. Using RNA sequencing, we analysed transcriptome‐wide differential gene expression from liver, head kidney and gonads, between male versus female AL and CR groups. We sampled the hind‐gut to genotype microbial composition using 16S rRNA amplicon sequencing to investigate potential sex‐specific changes induced by fasting.

We further compared individuals exposed to CR to young and old control individuals (respectively, 4 months and 3 years old) from the same stock populations to better understand the effect of CR on senescence and provide insights into how CR‐induced genetic and microbial modifications may influence aging and rejuvenation processes.

## 2 Material and Methods

### 2.1 Study Design and Organism

Three‐month‐old *Hippocampus erectus* juveniles from an aquarium‐bred population kept at the GEOMAR Helmholtz Centre for Ocean Research Kiel were used for the experiment. Fifty‐two juvenile seahorses were distributed across 18 tanks (20 L aquaria), with 16 tanks housing three seahorses each and two tanks (one for the AL treatment and one for CR treatment) housing two seahorses each.

The aquaria had a recirculating system with a temperature of 23°C–25°C, salinity 30–35 PSU, nitrate 20–50 mg/L, phosphate below 1 ppm, ozonisation and a 20% weekly water change. Filters, plastic seagrass and oxygen tubes were cleaned separately with hot water bi‐weekly; left‐over food or waste was suctioned and removed 30 min after feeding to prevent biofilm formation.

We allocated 26 individuals to an AL feeding regime and another 26 to a CR regime. The tanks were divided into nine AL and nine CR, with alternating tanks placed next to each other to reduce tank location effects. All fish were fed with defrosted mysids; the AL group was fed twice a day, in the morning (8 am) and in the late afternoon (4 pm); the CR group was fed every other day, that is, one day no food, the next day they were fed like the AL group. Seahorses show a secondary loss of stomach, requiring them to feed continuously for survival. They usually consume small crustaceans floating in the water, using their camouflage to remain still and ambush prey at the right moment with a swift strike (Woods 2002).

To ensure that observed effects in the CR group were due to calorie reduction and not nutrient deficiency, both groups were given vitamins. The supplements were provided equally on the days when both groups were fed and included 15 μL of Microbe‐Lift Garlic feed supplement (contains pure garlic, known to reduce bacterial diseases in fish (Bhatwalkar et al. 2021)), 15 μL of JBL Atvitol multivitamin drops for aquarium fish and half a capsule (730 mg) of Omega‐3 1000 (unsaturated omega‐3 fatty acids and vitamin E). These additives were left in the defrosted food for 2–3 min to ensure absorption. Seahorses were monitored daily during feeding for a health check and/or any abnormal swimming behaviours.

At the start of the experiment, all animals were weighed and measured for length before being placed in their respective tanks. For the first seven days, we gradually reduced the food in the CR group by feeding them only once in the morning on fasting days to minimise stress and facilitate habituation. Starting from day eight, the exact feeding regime was implemented, with the CR group receiving no mysids on alternate days. The study spanned a total of 157 days, which roughly equates to five months, during which pouch development was closely monitored to determine when sexual maturation was taking place and to ensure separation of males and females in same sex‐tanks to prevent mating. For growth monitoring, they were weighed in a small beaker with previously calibrated tank water, roughly every 50 days. Upon termination of the dietary experiment, the seahorses were euthanised with an overdose of MS‐222 (Tricaine methane sulfonate; 500 mg/L; Sigma‐Aldrich). They were weighed and measured for total body length (snout to tail). Additionally, female ovaries were weighed and the width and length of pouch were measured for males. Liver, head kidney and gonads for gene expression analysis were immediately preserved in RNA later stored at 4°C for 3 days, followed by long‐term storage at −20°C. Hind‐gut for microbiota genotyping was placed in a sterile Eppendorf tube and stored at −80°C.

To assess how CR influences aging compared to the AL treatment, we took 4‐month‐old juveniles labelled as control young (CY) and 3‐year‐old adults labelled as control old (CO) that had been kept in the same GEOMAR stocks. They were also euthanised with 500 mg/L of MS‐222, then measured for weight and body length and dissected to retrieve organs, which were stored as described above.

### 2.2 RNA and DNA Extraction, Library Preparation and Sequencing

RNA extraction of liver, head kidney and gonads was performed using the RNeasy Mini Kit from Qiagen (Venlo, Netherlands) according to the manufacturer's instructions. The concentration of the extracted RNA was measured using a Peqlab NanoDrop ND‐1000 spectral photometer (Erlangen, Germany), and the samples were subsequently stored at −80°C. One hundred and twenty samples of 40 randomly selected individuals (five males and five females from AL, CR, CO, CY, multiplied by the three tissues) were sent to BGI Tech Solutions in Hong Kong for library preparation and mRNA sequencing. For library preparation, the DNBSEQ Eukaryotic Strand‐specific mRNA library was utilised, and the sequencing of stranded mRNA was carried out on the DNBseq platform. The sequencing parameters included 150 bp reads and 25 M clean paired‐end reads per sample.

To extract the microbiota from the hind‐gut, DNA extraction was performed using the DNeasy Blood & Tissue Kit (QIAGEN, Germany) following the manufacturer's protocol, including a pre‐treatment for Gram‐positive bacteria with ameliorations (Korsch et al. 2018). Subsequently, a 16S PCR was conducted to verify the success of the extraction. The library preparation for a total of 57 individuals was performed by the Institute for Experimental Medicine (UKSH, Campus Kiel). This group included 37 seahorses from the dietary experiment with an additional 10 young and old individuals (5 AL, 11 CR, 5 CY, and 5 CO females, as well as 10 AL, 11 CR, 5 CY, and 5 CO males). Amplicon sequencing of the V3–V4 hypervariable region (341f/806r) was conducted using the Illumina MiSeq platform (Illumina, USA) with 2 × 300‐bp paired‐end read settings at the IKMB Kiel.

### 2.3 Data Analysis

#### 2.3.1 Morphology

In our experiment we complied stringently with daily animal welfare screening processes and seahorses were euthanised when scores were insufficient, resulting in a total of 38 individuals who survived until the conclusion of the experiment (day 157). Among them, the survivors encompassed 16 individuals belonging to the AL group with 11 AL males and 5 AL females, while 22 individuals belonged to the CR group with 11 CR males and 11 CR females. At the beginning of the experiment, we could not divide the seahorses by sex as they were not sexually mature, resulting in a smaller number of AL females compared to the other groups.

For the morphology analysis, we included only the 38 individuals that survived to the end of the experiment, with an additional separate analysis comparing the diet groups to the young and old age groups of seahorses (5 CY, 5 CO both sexes). All the statistical analyses were done in Rstudio (v.4.2.2) (R Core Team 2022).

**FIGURE 1:**
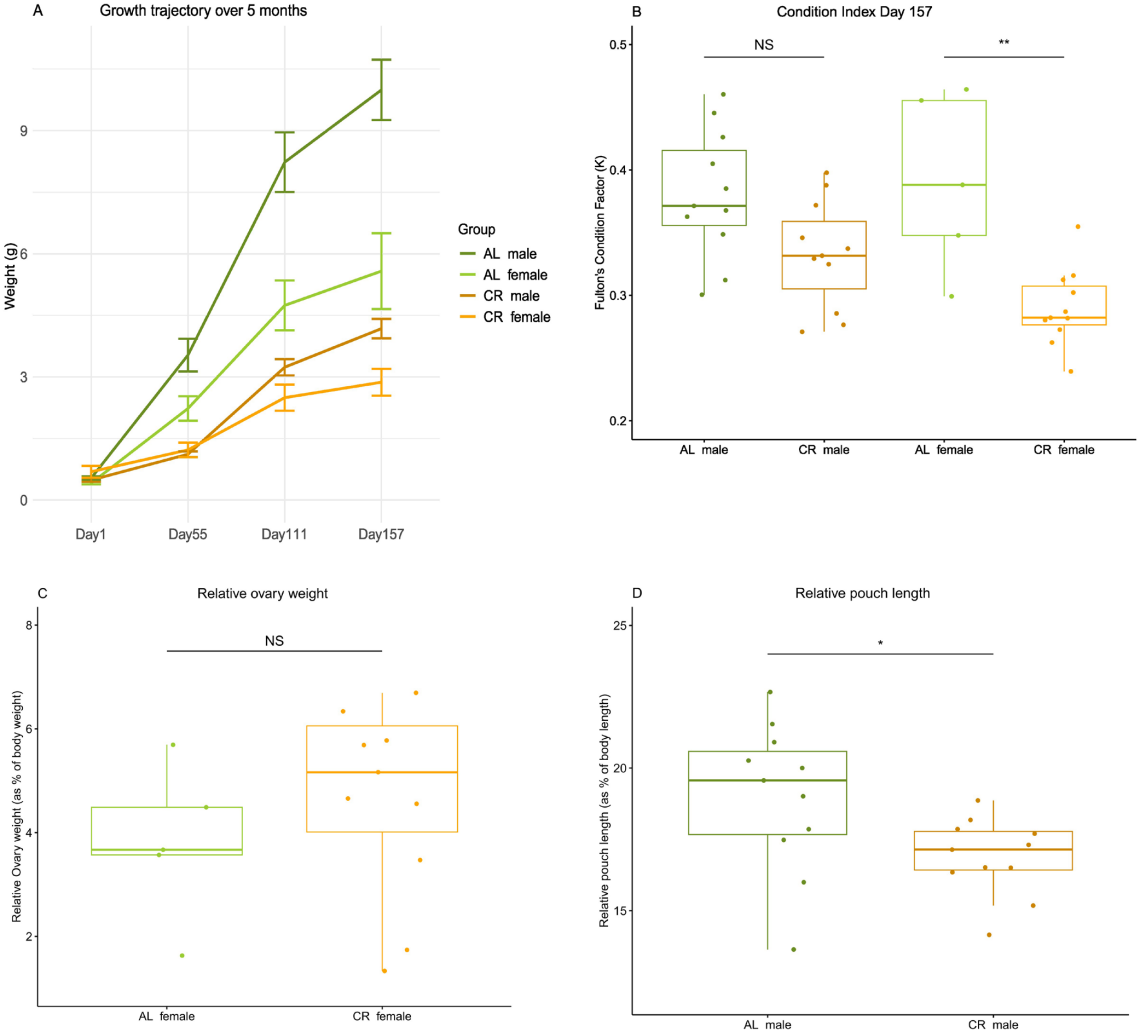
Effects of caloric restriction (CR) and ad libitum (AL) diets on growth, condition and reproductive morphology in male and female seahorses. (A) Growth trajectories of male and female seahorses under CR and AL diets over five experimental months. The y‐axis represents weight (grams), and the x‐axis represents time with four measurement time points. AL males (dark green), AL females (light green), CR males (brown), and CR females (orange) are shown, with error bars indicating standard errors. (B) Condition index at the end of the experiment, comparing Fulton's condition factor (weight/length^3^) across sexes and dietary treatments. Significant differences are denoted as *p < 0.05, **p < 0.001, and ***p < 0.0001. (C) Relative ovarian weight of CR and AL females, expressed as a percentage of body weight. The y‐axis shows relative ovarian weight (%). (D) Relative pouch length in male seahorses from CR and AL treatments, expressed as a percentage of body length. The y‐axis shows relative pouch length (%).

We tested various morphological parameters for both dietary treatments (AL, CR) and age control groups (CO, CY) on male and female fish, including weight (g) and total body length (cm), as well as Fulton's condition factor (weight/length^3^) to better assess overall fish condition (Robinson et al. 2008). Additionally, we calculated relative male pouch length (pouch length/total body length × 100) (Figure 1D and Figure S1H) and relative ovary weight (ovary weight/total body weight × 100) (Figure 1C and Figure S1G). Normality was assessed using a Shapiro–Wilk test. Depending on the results, we applied either a Kruskal–Wallis test (KW‐test), followed by a Dunn's post hoc test, or a two‐way ANOVA followed by a Tukey Honest Significant Difference (Tukey HSD) test ((Treatment × Sex), Table S6 for statistics). Furthermore, since weight measurements were taken at four time points (Days 1, 55, 111 and 157), we analysed growth trajectories over time using a linear mixed model, with individuals as a random effect: lmer(weight (g) ~ Time × Treatment × Sex + (1|Ind), data = data). Pairwise comparisons for Treatment × Sex at each time point were conducted using *emmeans* (Table S6, Figure 1A).

#### 2.3.2 Differential Gene Expression Analysis

**FIGURE 2:**
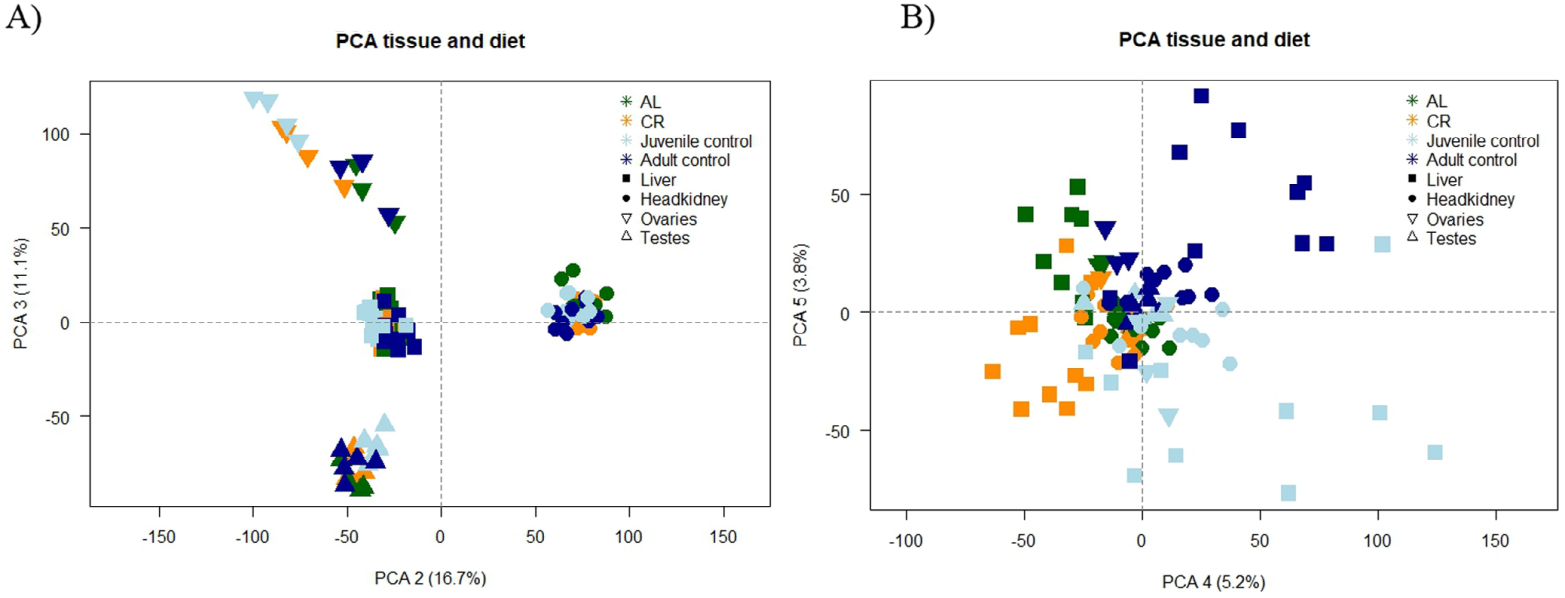
Principal component analysis (PCA) plots depicting organ separation and clustering of treatment groups. (A) PCA plot of PC2 (16.7% variance) and PC3 (11.1% variance). PC2 separates the head kidney from the liver and gonads, while PC3 further differentiates male and female gonads. Groups are colour‐coded as follows: CR (orange), AL (green), juvenile control (light blue) and adult control (dark blue). Organs are represented by different symbols: Liver (square), head kidney (circle), ovaries (downward triangle) and testes (upward triangle). (B) PCA plot of PC4 (5.2% variance) and PC5 (3.8% variance). PC4 separates treatment groups and age cohorts, while PC5 distinguishes CR and CY from AL and CO.

The fasting effects were characterised by constructing differential expression signatures associated with each organ. The integrity and quality of the RNA Illumina sequencing results from BGI Tech Solution were thoroughly assessed and controlled using FastQC (v.0.11.9) (Andrews 2010). The adapters had already been trimmed by BGI Tech Solutions. Regrettably, during the run, six samples failed, all of which were ovarian tissue (two AL, one CR, two OC, and one YC). For the alignment process, we used STAR (v.2.7.9) (Dobin et al. 2013) to map the reads to a genome assembly of *Hippocampus erectus* (BioProject: PRJNA347499). Subsequently, read counts were obtained using TPMCalculator (Alvarez et al. 2019). In order to investigate potential distinctions among treatment groups (AL, CR, CO, CY) within specific organs, we partitioned the dataset by organ type. On logarithmically transformed count data, we employed the “adonis2” function and the “bray” method and conducted a PERMANOVA analysis (Permutational Multivariate Analysis of Variance Using Distance Matrices). Our examination evaluated the influences of diet and sex in the liver, head kidney, ovaries and testes. We did a principal component analysis (PCA) with multiple PCs to visualise group clustering and possible outliers (Figure 2 and Figure S2). For differential gene expression (DGE) analysis, we first used the edgeR package (v.3.40.2) (Robinson et al. 2010) to scale the count data to counts per million (cpm) and filtered it based on a minimum threshold of 10 counts in at least 5 libraries. We performed this process separately for each organ, resulting in different gene sets for each: 13,015 genes for the liver, 16,470 genes for the head kidney, 17,411 genes for the testes, and 16,897 genes for the ovaries. To address composition biases, we normalised the data using the trimmed mean of values (TMM) with the calcNormFactors function. This step calculated normalisation factors for each sample, effectively eliminating composition biases between libraries. Next, we utilised the limma package (v.3.54.1) (Ritchie et al. 2015) for DGE analysis. Limma is a linear model‐based method that employs an empirical Bayes approach and the voom method to convert count data to a continuous scale accounting for batch effects and reducing technical variation. The voom method is well‐suited for handling library sizes that vary significantly (Law et al. 2014). To compare several groups, we created a matrix of independent contrasts, enabling us to perform a one‐way analysis of deviance (ANODEV) for each gene. Subsequently, we estimated coefficients of interest between groups to determine log fold changes using the “contrasts.fit” function. We applied empirical Bayes moderation to shrink the estimated variance and performed a moderated *t*‐test to identify differentially expressed genes (DEGs). For further downstream analysis, we focused only on adjusted *p* values below 0.05 and corrected for multiple testing with Benjamini–Hochberg (BH) correction.

#### 2.3.3 Gene Ontology Analysis

Gene ontology (GO) analysis was performed using the g:Profiler web tool (accessed on July 6, 2023). This tool enabled us to conduct gene enrichment analysis by comparing the significantly DEGs between AL and CR seahorses across the different organs. To annotate the genes of *Hippocampus erectus* , we employed OrthoFinder and conducted a homology‐based search using *Danio rerio* (GRCz11) as a reference species. Adjusted statistical significance was set for “g:SCS threshold” value at 0.05, focusing exclusively on annotated genes. We treated numeric IDs as “ENTREZGENE_ACC” and limited the data sources to GO biological processes (BP), as well as biological pathways from the Kyoto Encyclopaedia of Genes and Genomes (KEGG) and Reactome (REAC) databases. To enhance the specificity of our analysis, we applied a maximum term size of 1000 to exclude overly broad categories and prioritise more informative results.

#### 2.3.4 Microbiome Data Analysis

The 16S rRNA amplicon sequencing analysis was performed via the QIIME2 platform (v.2022.8.3) (Bolyen et al. 2019). With dada2, the raw paired‐end Illumina reads were demultiplexed, and amplicon primers for the 16S V3‐V4 region were trimmed; quality was controlled by “denoising” sequences (in order to better discriminate between true sequence diversity and sequencing errors). Low‐quality reads were filtered (manual trimming at forward read at 250 bp and reverse at 210 bp), forward and reverse samples were merged, and possible chimeras were removed. A phylogenetic tree was constructed using “Fasttree” (v.2.1.11) that infers approximately maximum likelihood based on the longest root (Price et al. 2009). Interactive α rarefaction curves were computed for max_depth of 20,000 to determine whether the samples were sequenced deeply enough to capture all the bacterial community members. Samples were classified for the V3/V4 hypervariable region based on taxonomic groups using the SILVA v138 database and a naïve Bayes based classifier (Yilmaz et al. 2014). The amplicon sequence variants (ASV) were filtered to remove sequences from chloroplasts and mitochondria and exported at genus level as an operational taxonomic unit (OTU) for further analysis in RStudio (v.2022.07.2). To reduce noise, we applied a filtering threshold, including only OTUs that were present in at least three samples across the entire dataset. The remaining samples consisted of 5 AL, 11 CR, 5 CY, and 5 CO females, and 10 AL, 11 CR, 4 CY, and 4 CO males. OTU counts were normalised across samples to ensure data comparability.

α‐diversity was measured using the Shannon index to measure diversity within individual seahorses using the vegan package (v.2.6.4) (Oksanen et al. 2020). β‐diversity was assessed using the Bray Curtis dissimilarity matrix (based on relative abundance) to measure differences between the individual group of seahorses (vegdist function). Hypothesis testing for both α‐diversity was done with the Kruskal–Wallis test followed by a post hoc Dunn test (Table S6), while β‐diversity was performed using PERMANOVA with both diet and sex as factors (x ~ diet × sex, data = data, permutations = 999), preceded by testing for normality with a Shapiro–Wilk normality test. To compare groups, pairwise.adonis2 v0.4 (Arbizu and Arbizu 2020) was used as a post hoc test, with Benjamini–Hochberg (BH) correction for *p*‐value adjustment to account for multiple testing. To visualise the results, we used boxplots to display α‐diversity and non‐metric multidimensional scaling (NMDS) for β‐diversity. For the NMDS, we employed 95% confidence ellipses to represent group variability, with the choice of ellipses based on standard error due to the high variance observed in the β‐diversity data. This approach allows for a clearer representation of group dispersion while accounting for the inherent variability in the data. We examined the OTUs using the *envfit* function from the vegan package (v.2.6.4) to fit the OTUs to the NMDS ordination space and calculate the correlation between the ordination scores of OTUs and the treatment variables. By employing permutation tests with 999 iterations, we obtained *p*‐values to assess the significance of these correlations. We plotted the OTUs that significantly explained the NMDS Bray‐Curtis ordination (*p*.val < 0.001).

Relative abundance was calculated for the phylum and genus taxonomic level (Figure 4A and Figure S4B). The genus *Vibrio* (family *Vibrionaceae*) exhibited the highest frequency and variation across samples (Figure 4A and Figure S4B). Given the dominance of *Vibrio*, we sought to examine the broader background microbial community by focusing on less abundant taxa. To achieve this, we conducted an additional analysis excluding *Vibrio*, renormalised the data and applied a centred log‐ratio (CLR) transformation to convert relative abundances into log‐ratio values. This transformation mitigates the unit sum constraint, allowing for more accurate comparisons across samples. Following this pre‐processing, all statistical tests were repeated (Table S6), and alpha and beta diversity metrics were recalculated.

#### 2.3.5 Correlation Analysis Between Gut Microbiota and Transcriptomic Data

To assess potential associations between microbiota composition and host transcriptomic profiles, we performed a Mantel test comparing the 205 bacterial genera identified in our 16S rRNA sequencing data with the normalised transcriptomic data from liver, head kidney, testes and ovaries (Table S6). The Mantel test evaluates correlations between two distance matrices, using the Bray–Curtis dissimilarity method to assess the relationship between microbiota and gene expression patterns. Significant correlations were further explored using multidimensional scaling (MDS), an ordination method that reduces high‐dimensional data to a lower‐dimensional space for visualising potential associations between expressed genes and bacterial taxa. To further investigate the functional implications of the significant associations (*p* < 0.01), we performed a gene set enrichment analysis using the g:Profiler web tool to identify enriched pathways related to the associated genes. The results of this analysis are provided in Supporting Information, Section 3.6. Additionally, we conducted a correlation analysis specifically focused on immune‐related genes and their association with bacterial taxa. The resulting associations are presented in Figure S5.

## 3 Results

### 3.1 Effects of Caloric Restriction on Growth and Condition Index

The reduced caloric intake significantly impacted growth in the CR group (Figure 1). By the end of the experiment, the AL group had a larger overall size and was heavier compared to the CR group (Figure 1A and Figure S1A–D, KW‐test *p* < 0.0001). Prior to beginning the diet treatment, the seahorses had a mean weight of 0.58 ± 0.08 g for CR and 0.50 ± 0.03 g for AL (LMM *p* = NS, Table S6) and a mean length of 4.5 ± 0.21 cm for CR and 4.6 ± 0.21 cm for AL (Figure S1C, LMM *p* = NS, Table S6). After 157 days, the AL group had a mean weight of 8.61 ± 0.2 g, while the CR group displayed a mean weight of 3.51 ± 0.24 g (Figure 1A, LMM *p* < 0.001). Across the growth trajectory monitoring, we noticed already on day 55, weight measurements showing stark contrasts for male seahorses with AL 3.53 ± 0.4 g versus CR 1.12 ± 0.07 g (LMM adj. *p* < 0.001, Figure 1A) and then for both sexes on day 111 with AL male 8.2 ± 0.73 g versus CR 3.24 ± 0.19 g (LMM adj. *p* < 0.0001, Figure 1A), and AL female 4.73 ± 0.61 g versus CR 2.5 ± 0.32 g (LMM adj. *p* < 0.001, Figure 1A, Table S6).

After 5 months, the differences between males and females became even more pronounced. Female seahorses exhibited a slight but significant difference in weight, with AL females weighing 5.6 ± 0.9 g compared to 2.8 ± 0.33 g for CR females (Dunn‐test, adj. *p* < 0.02, Figure 1A and Figure S1B). In contrast, males displayed a more substantial and highly significant weight difference, with AL males weighing 9.9 ± 0.73 g versus 4.18 ± 0.24 g for CR males (Dunn‐test, adj. *p* < 0.0004, Figure 1A and Figure S1B). However, when we calculated the condition index, we observed that the CR treatment had a smaller impact on the overall condition of males (Tukey HSD, adj. *p* = 0.09) compared to females (Tukey HSD, adj. *p* < 0.002, Figure 1B). It is important to interpret the results for female seahorses with caution, as only five AL females remained by the end of the experiment compared to 11 CR. This difference is most likely due to the initial unequal sex ratio, rather than mortality, as both male groups maintained 11 individuals each.

Throughout the dietary period, we closely monitored the fish and documented the emergence of pouch formation in male seahorses, as they were not sexually mature at the beginning of the experiment. On day 19, we observed the first signs of pouch formation in an AL, and by day 28, we were able to distinguish between males and females in all AL seahorses. On day 39, pouch formation was documented in the CR group, and by day 47, we could confidently determine the sex of all CR individuals. Ovarian weight measured at the end of the fasting experiment did not significantly differ between AL and CR groups (ANOVA, *p* = 0.3; Figure 1C). This lack of significance could be attributed to the lower number of biological replicates in the AL group (*n* = 5) compared to the CR group (*n* = 11). Furthermore, at the end of the experiment (day 157), we assessed the relative male pouch size between treatment groups (Figure 1D and Figure S1H). AL males showed a moderate difference, with pouch size averaging approximately 19% of total body length, compared to 17% in CR males (ANOVA, *p*‐value < 0.03, Figure 1D).

In the Supporting Information, we also compare the condition index of the young and old control groups (Figure S1E–G, Table S6 statistics). Specifically, we compared the condition factor between CO and CY for both males and females (Figure S1E), and further compare the condition factor between the dietary treatment groups AL and CR with the age control groups CO and CY (Figure S1F). Due to differences in life stages between the CR experiment and the age control groups, direct comparisons are not fully appropriate, and therefore this analysis is discussed only in the Supporting Information. However, we found no significant difference in fish condition between AL and CO (Tukey HSD, adj. *p* = 0.9), a moderate difference between CR and CO (Tukey HSD, adj. *p* = 0.03) with a rather more significant effect when comparing CR and CY (Tukey HSD, adj. *p* = 0.007), but a very strong significant difference between AL and CY (Tukey HSD, adj. *p* < 0.00001, Figure S1F).

### 3.2 Effects of Caloric Restriction on Tissue Composition

RNA sequencing (RNAseq) of the liver, head kidney, and gonads (testes and ovaries) was analysed to assess sex‐specific effects of CR treatment on metabolic, immunological, and reproductive acclimatisation. PERMANOVA analyses of the five‐month CR and AL treatments revealed that the effects of treatment and sex varied across tissues. In the liver, both treatment and sex had significant effects (PERMANOVA; *p* < 0.001), but no interaction was observed (Table S6). Pairwise comparisons with Bonferroni correction identified significant differences between CR and AL males, AL females and AL males, and AL females and CR males (adj. *p* < 0.05). Notably, the treatment effect was weaker in females, as no significant difference was found between AL and CR females. In the head kidney, the treatment effect was not as pronounced (*p* < 0.04); instead, a strong sex effect was observed (*p* < 0.001), with the only significant interaction represented between CR females and AL males (*p* < 0.05). Reproductive tissues showed no strong response to CR, with no effect on testes (*p* = 0.6) and a non‐significant trend in ovaries (*p* = 0.06, Table S6).

To investigate whether fasting induces physiological rejuvenation, we compared gene expression profiles of seahorses subjected to different feeding regimens (AL, CR) with those of control age cohorts (CO, CY). PERMANOVA tests revealed significant differences between dietary treatments and age groups across all tissues (*p* < 0.002), except for the testes (*p* = 0.2). In the liver, AL seahorses differed significantly from CY (adj. *p* = 0.006) and to a lesser extent from CO (adj. *p* = 0.012). Interestingly, the opposite pattern was observed for CR seahorses, with CR versus CO (adj. *p* = 0.006) and CR versus CY (adj. *p* = 0.012) showing reversed significance trends (Table S6). In the head kidney, AL seahorses again differed significantly from CY (adj. *p* = 0.006) and barely reached significance with CO (adj. *p* = 0.048). However, unlike the liver, CR seahorses displayed a significant difference from CY (adj. *p* = 0.006) but were less distinct from CO (adj. *p* = 0.024). As previously noted, the male testes exhibited no significant differences between treatment groups or age cohorts (Table S6). While a PERMANOVA detected an overall significant effect in the ovaries, none of the pairwise comparisons remained significant after correction.

The PCA analysis, comparing all organ types and dietary treatments, revealed that the first three principal components (PCs) were primarily driven by tissue and organ effects, without clear treatment‐related differences (Figure S2 and Figure 2A). Specifically, PC1 separated the liver from the other organs (Figure S2), PC2 distinguished the head kidney from the liver and gonads (Figure 2A), and PC3 further separated male and female gonads (Figure 2A). In contrast, PCs 4 and 5 were more influenced by dietary and age‐related factors (Figure 2B). PC4 separated the dietary groups (AL and CR) from the age cohorts (CO and CY), while PC5 distinguished CR and CY from AL and CO (Figure 2B). These differences were most pronounced in the liver, where the separation was particularly clear (Figure 2B).

We next explored the gene expression profiles to further investigate the effects of CR on seahorse physiology, focusing on sex differences and aging. The DGE analysis revealed considerable heterogeneity in the number of significantly differentially expressed genes across different organs (Table S1) and reflected the statistical results above. Females exhibited a stronger transcriptional response to caloric restriction (CR) in the liver, with 375 DEGs compared to 83 in males (Table S1). Sex differences were more pronounced in the AL group (501 DEGs between males and females) but were reduced under CR (83 DEGs), indicating a convergence in gene expression profiles between sexes under dietary restriction. For the age cohort comparisons, male CR induced a stronger shift in gene expression compared to AL, particularly when both treatments are compared to the same age cohort (e.g., CR vs. CO = 2454 DEGs vs. AL vs. CO = 933 DEGs). In contrast, in females, the difference between CR and AL is less pronounced, and CR appears to buffer age‐related changes more effectively, as indicated by the much lower number of DEGs in CR versus CY (601) compared to AL versus CY (1891).

The head kidney exhibited pronounced sex differences in gene expression but showed a minimal response to caloric restriction. In females, there were no DEGs between AL and CR, indicating that dietary restriction did not significantly alter gene expression in this organ. In males, only four DEGs were detected between AL and CR, reinforcing the weak effect of caloric restriction (Table S1). However, the sex differences were striking—AL males and females differed by 973 DEGs, and this difference increased further under CR (1728 DEGs). When comparing dietary treatments across age cohorts, younger males exhibited the strongest transcriptional response in the head kidney. AL males showed almost no difference from old controls (11 DEGs) but had a substantial response when compared to young controls (1674 DEGs). Similarly, CR males showed a moderate shift compared to old controls (801 DEGs), which became much more pronounced in comparison to young controls (2846 DEGs). In females, these effects were weaker but followed a similar trend, with 502 DEGs (AL vs. old controls) and 632 DEGs (AL vs. CY), while CR resulted in 558 DEGs (vs. CO) and 1054 DEGs (vs. CY).

Although CR did not affect the male testes, the female ovaries exhibited 366 DEGs between the AL and CR treatments. When comparing the age cohorts, no significant differences were found between AL and CO or CR and CY. In contrast, there were 218 significant DEGs between CR and CO, while a striking 4966 DEGs were found between AL and CY.

### 3.3 Gene Enrichment Analysis Reveals Sex‐Specific Fasting Effects on Liver Metabolism

In the female liver (AL vs. CR), the most significantly enriched pathways across GO:BP, KEGG and REAC analyses were related to sterol and cholesterol biosynthesis (Table S2, sheet A). Genes upregulated in CR females included cytochrome P450 family 51 (*cyp51*), squalene epoxidase (*sqle*) and mevalonate kinase (*mvk*), all crucial in cholesterol and sterol biosynthesis (Cirmena et al. 2018). Other upregulated genes, such as hormone‐sensitive lipase (*lipe*) and lipase hepatic A (*lipca*), play key roles in lipid metabolism and uptake (Table S3) (Santamarina‐Fojo et al. 2004). CR also upregulated genes involved in ATP production, oxidative stress response, and autophagy, including isocitrate dehydrogenase (*idh2*), peroxiredoxin 4 (*prdx4*), peroxiredoxin 6 (*prdx6*), autophagy‐related 13 homologue (*atg13*) and acetoacetyl‐CoA synthetase (*aacs*), all of which contribute to energy homeostasis and survival under nutrient depletion (Reitman and Yan 2010; Rhee 2016; Bergstrom 2023; Tian et al. 2009).

Conversely, CR downregulated genes related to steroid synthesis, including P450 family 17 subfamily A (*cyp17A2*) and the progesterone receptor (*pgr*) (Uno et al. 2012), as well as genes involved in iron metabolism and erythropoiesis, such as STEAP3 and erythropoietin receptor (*epor*) (Ohgami et al. 2006; Rossert and Eckardt 2005).

**FIGURE 3:**
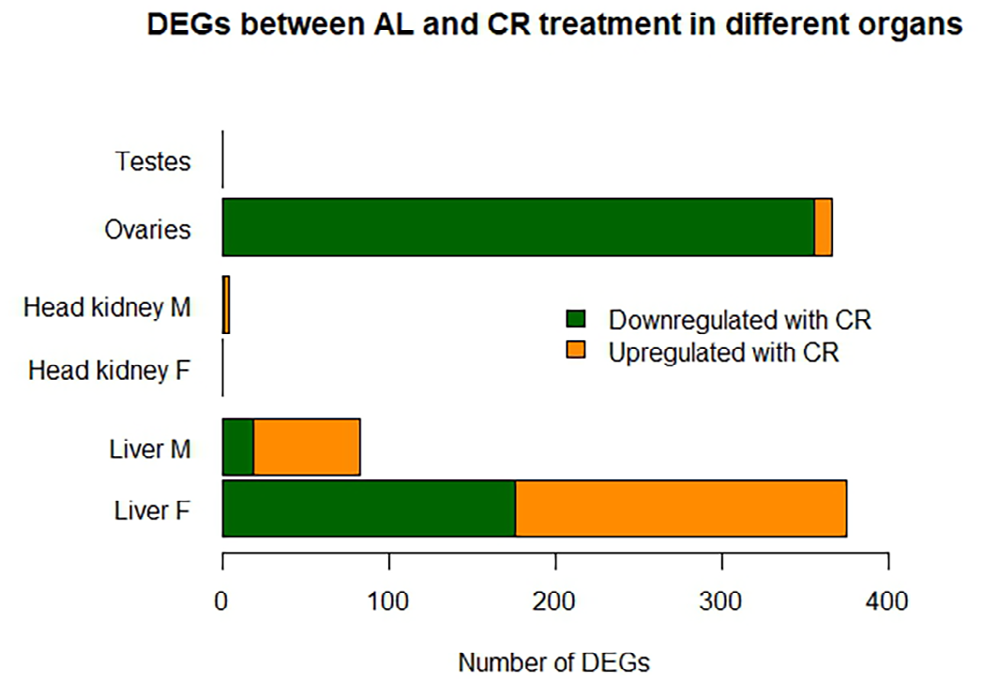
Sex‐specific differential gene expression in AL and CR‐fed seahorses across organs. Bar plot showing differentially expressed genes (DEGs) between AL and CR‐fed seahorses across various organs, categorised by sex (M = male, F = female) on the y‐axis. Genes upregulated in the AL treatment (downregulated in CR) are shown in green, while genes upregulated in CR (downregulated in AL) are shown in orange. The x‐axis represents the number of DEGs with a significant adjusted *p*‐value (< 0.05), corrected using the BH method. Additionally, Figure S3 presents another bar plot depicting sex‐biased gene expression in AL and CR‐fed seahorses.

In males, DEGs in the liver (AL vs. CR) were primarily associated with energy production, with enriched pathways including ATP biosynthetic processes (GO:BP), oxidative phosphorylation (KEGG) and mitochondrial biogenesis (REAC) (Table S2, sheet B). Although CR affected fewer genes in males than in females, most were upregulated in fasted individuals (Figure 3). The few downregulated genes included *coq8ab*, involved in coenzyme Q biosynthesis for mitochondrial electron transport, and *bco11*, which cleaves beta‐carotene into retinal, a precursor of vitamin A (Helgeland et al. 2019). Some upregulated genes overlapped with those in females, such as *idh2* and *lipca* (Table S2, sheet B). However, CR males showed increased expression of *ldhd*, which converts pyruvate to lactate and regenerates NAD+, and *fetub*, a glycoprotein linked to impaired glucose metabolism and insulin resistance (Meex et al. 2015). Additional upregulated genes included *thop1*, which regulates energy metabolism, with knockout studies linking it to increased oxidative metabolism (Ferro et al. 2020), and *selenop2*, a selenoprotein involved in oxidoreductase reactions (Lei et al. 2022). We also identified DEGs related to circadian rhythm regulation that were induced in expression in the liver of CR males, including clock circadian regulator b (*clockb*) and cryptochrome circadian regulator 3 (*cry3a*). The circadian rhythm is closely linked to the daily cycle of nutrient intake and metabolic control. CLOCK, a key transcription factor in the circadian clock machinery, modulates the chromatin environment by altering histone acetyltransferase (*HAT*) activity (Doi et al. 2006). *Cry3a*, an ortholog of human *cry1*, functions as a molecular clock regulator by participating in an autoregulatory transcriptional loop with an ~24‐h periodicity, where CLOCK binds to the cry1 promoter region (Sancar 2004).

### 3.4 Calorie Restriction Reduces Immune Response and Lipid Metabolism Pathways in Ovaries

No significantly DEGs were found in the testes between AL and CR treatments, compared to ovaries which appeared far more affected through the diet with 356 genes being downregulated under CR and only ten upregulated (Figure 3). Enrichment analysis highlighted key pathways, including lipid catabolic process (GO:BP), lysosome (KEGG), and innate immune system (REAC) as the most enriched (Table S2, sheet C). Among the downregulated genes in CR, several were involved in lipid catabolism, such as ATPase H+ transporting V1 and V0 (*atp6v1f, atp6v1e1b, atp6v0cb*), as well as nutrient‐sensing genes that regulate longevity pathways. These included forkhead box O1a (*foxo1a*), a transcription factor that integrates insulin action with nutrient and energy homeostasis, part of the FOXO family regulating metabolism, stress resistance, apoptosis, and longevity (Cheng and White 2011). Similarly, sestrin 2 (*sesn2*), known to activate mTORC1 (Wolfson et al. 2016), and TELO 2 interacting protein 2 (*tti2*), which stimulates downstream cascades for cell growth via mTORC1 (Brown and Gromeier 2017), were also downregulated. The downregulation of these genes in CR suggests a suppression of growth and cell proliferation pathways. Conversely, similar pathways were upregulated in AL ovaries, including genes like claudin 7 (*cldn7b*), involved in solute transfer across cell monolayers and possibly contributing to cancer cell survival, growth, and invasion (Dahiya et al. 2011), and Rhophilin Rho GTPase binding protein 2 (*rhpn2*), a scaffold protein that participates in protein complex assembly, intracellular signalling, and endosome targeting (Steuve et al. 2006).

Immunological responses in the ovaries of CR females were suppressed, with multiple genes related to the innate immune system and lipid metabolism showing downregulation. Notably, two acyl‐CoA synthetase (*ACS*) enzymes, ACS bubble gum (*acsbg*) and long‐chain ACS (*acsl*), are both involved in lipid metabolism. *Acsl*, in particular, is known to play a role in immune response in rainbow trout, being upregulated under hypoxic conditions to enhance tolerance (Lopes‐Marques et al. 2018; Ma et al. 2022). Alongside these lipid‐metabolism‐related genes, all genes involved in the innate immune pathway were also downregulated in CR ovaries (Figure 3, Table S3 sheet D). This included genes such as cytokine‐like interleukin 12 receptor beta 2a (*ilrb2a*), which stimulates interferon‐γ production from T cells and NK cells (Fujiwara et al. 2003); M17, a subfamily cytokine with similarities to mammalian IL‐6 that induces macrophage activation in goldfish (Hanington and Belosevic 2007); and *mpeg1.2*, an antibacterial gene induced by infection (Benard et al. 2015). Other downregulated genes in this pathway included interferon regulatory factor 5 (*irf5*), involved in antiviral and inflammatory responses (Xia et al. 2012), chemokine receptors (*cxcr4a, cmklr1*), and tumour necrosis factor (tnf), all contributing to the inflammatory response (Mokhtar et al. 2023). Furthermore, toll‐like receptors (TLRs) 21 and 22, essential pattern‐recognition receptors for detecting pathogen‐associated molecular patterns (PAMPs), were also downregulated.

### 3.5 Calorie Restriction Promotes Greater Gut Microbial Diversity

**FIGURE 4:**
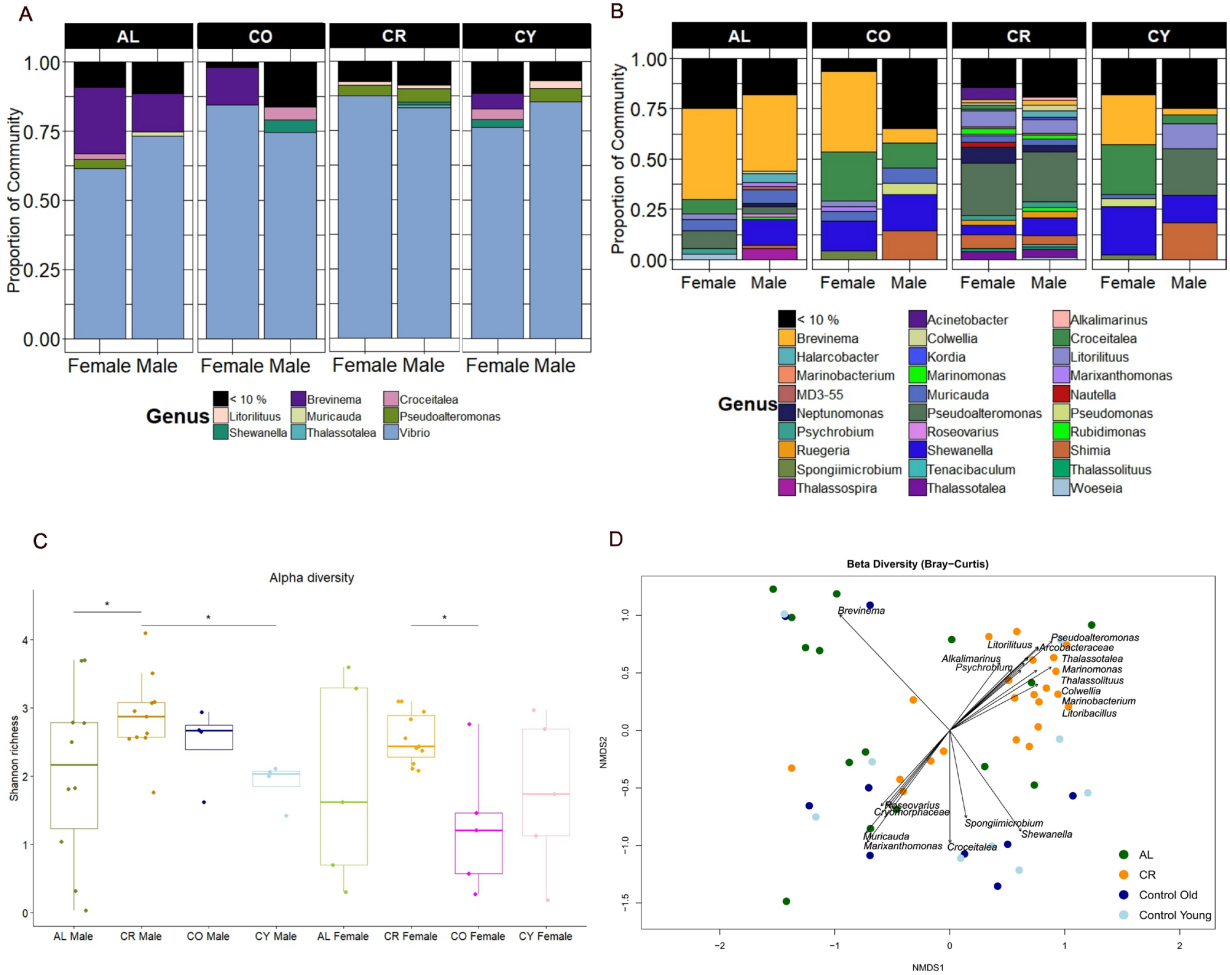
Microbial biodiversity analysis from hindgut tissue of seahorses. (A) Bar plot showing the relative abundance of microbial taxa at the genus level. Panels are categorised by diet—ad libitum (AL) and caloric restriction (CR)—as well as by age group—control old (CO) and control young (CY). Within each subcategory, data are further divided by sex (male and female). Low‐abundance taxa (< 10%) are grouped together. (B) Bar plot showing the relative abundance of microbial taxa at the genus level after excluding *Vibrio*, allowing for better visualisation of background microbial communities. (C) Boxplots of alpha diversity (Shannon index) for diet groups (AL and CR) and age groups (CO and CY), separated by sex. *p*‐values are derived from the post hoc Dunn test (see Table S6); significance is indicated with * as *p* < 0.05. (D) Non‐metric multidimensional scaling (NMDS) plot based on the Bray–Curtis dissimilarity metric, comparing microbial communities across diet and age groups, with each dot representing an individual. Arrows indicate microbial taxa significantly associated with community structure (*p* < 0.01), highlighting key indicator species for each group.

The analysis of microbial 16S rRNAseq in seahorse hind‐gut tissue found that *Vibrio* was the most abundant bacterial genus across all treatments (CR, AL), age cohorts (CO, CY) and sexes. However, its prevalence differed between treatments. In the CR group, *Vibrio* dominated the microbial community, comprising approximately 90% of the total abundance in females and 85% in males, while in the AL group, it accounted for a lower proportion, ranging from 60% in females to 75% in males (Figure 4A). This suggests that CR creates an environment where *Vibrio* outcompetes other bacterial taxa, whereas in the AL treatment, microbial diversity appears to be higher, allowing for the presence of additional genera.

To better understand the composition of smaller bacterial communities, we reanalysed the data after excluding *Vibrio*, re‐normalising, and applying CLR transformation to correct compositional biases, allowing for more accurate comparisons of less abundant taxa across all groups. This revealed a substantial presence of other taxa across all groups. In the AL treatment, *Brevinema* (family *Brevinemataceae*) emerged as the dominant genus accounting for approximately 45% of the relative abundance in females and 38% in males (Figure 4B). In fact, β‐diversity NMDS plots comparing treatment groups (AL, CR) and age cohorts (CO, CY) indicated a strong association between *Brevinema* and AL seahorses (Figure 4D). In contrast, CR seahorses exhibited a more diverse background microbial community, though with considerably lower relative abundances of individual taxa (Figure 4B). Among the most prominent bacterial groups in CR seahorses were several genera within the phylum Proteobacteria, including *Pseudoalteromonas*, *Neptunomonas*, *Litorilituus*, *Shewanella*, *Marinobacterium*, *Marinomonas*, and *Thalassotalea* (Figure 4B,D).

Shannon diversity analysis showed no significant differences in within‐individual variability between treatment groups (CR, AL) or age cohorts (CO, CY), with overall low richness values ranging from below 1 to a maximum of 2.5 (Levene's test, *p* > 0.05; Figure S4A, Table S6). This aligns with the dominance of *Vibrio* across all samples. However, when *Vibrio* was excluded, microbial diversity varied more within Al seahorses (Levene's test, *p* < 0.002, Table S6), also for CO and CY females, whose Shannon richness ranged from below 1 (indicating few dominant taxa) to 3 (indicating higher diversity with several coexisting species). In contrast, most CR individuals had more consistent values around 3 (Figure 4C). CO and CY males also exhibited higher diversity, averaging around 2–2.5. Significant differences in α‐diversity were detected between several treatment groups (Table S6), including CR males versus both AL and CY males (Dunn test, *p* = 0.04 and *p* = 0.01 respectively), and CR females versus CO females (*p* = 0.02). No significant differences were detected between sexes within the same treatment group, and only CO male versus CO females barely reached significance (*p* = 0.053). However, significant differences were observed in comparisons where treatment, sex, and age effects were confounded, such as between CR males and CO females or CY females and CR males (Table S6).

The effect of CR on β‐diversity, which measures differences in microbial community composition between groups, was the strongest driver of variation in our analysis. Significant differences were observed between CR and AL when analysing the full dataset, including *Vibrio* (adj. *p* < 0.006; Figure S4C). However, this effect was even more pronounced when examining background microbial communities, excluding *Vibrio* (adj. *p* < 0.002; Figure 4D). Additionally, significant differences emerged between CR and all other groups (AL, CO and CY) in the absence of *Vibrio* (adj. *p* < 0.002). In contrast, there was no effect of sex or sex by treatment interaction on gut microbiome composition (Figure S4D).

While no significant differences were observed in overall α‐ and β‐diversity measures between males and females, closer observation of the relative abundance results (Figure 4B) revealed some interesting trends in community composition across the different treatment groups for sex. For instance, *Brevinema* was similarly highly prevalent in both sexes in the AL group, compared to a lower abundance for both sexes in the CR group, while in CO and CY, females had a higher relative abundance compared to males. In contrast, *Croceitalea* was found to be dominant in female seahorses in the CO and CY groups, where it represented around 25% of the total microbiome composition. This dominance was notably lower in AL females (around 6%) and nearly absent in CR females. *Marinobacterium* was more abundant in CO and CY males (15%) but barely present in females, and showed low levels in both sexes in CR (ca. 5%). Finally, *Shewanella* was present in all groups and both sexes, except in AL females, where it was absent. These taxon‐level shifts highlight the complex interaction between diet, sex and microbiome composition.

## 4 Discussion

Beneficial health effects of CR are well‐documented across various model species (yeast, nematodes, fruit flies and rodents), with positive effects encompassing improved metabolism, reduction in inflammation, and a higher diversity of the gut microbiome (Moatt et al. 2016; Sinclair 2005). This has led to the belief in evolutionarily conserved mechanisms of CR effects across the animal kingdom, which were challenged by a meta‐analysis, revealing twofold stronger effects in model species than in non‐model species (Nakagawa et al. 2012). This is potentially attributed to genetic sensitivity and publication bias favouring positive results in model organisms. Such bias underscores our constrained comprehension of how fasting impacts non‐model systems, which exhibit different evolutionary adaptations to food availability and varied evolutionary flexibility in reproductive systems, involving female‐and male‐specific resource allocation trade‐offs (Kane et al. 2018; Moatt et al. 2016). These differences could shape how males and females adjust to nutritional stress, leading to divergent fasting effects across species.

Our study aimed to elucidate the effect of CR, implemented through an alternating fasting regimen, on *Hippocampus erectus* . In this non‐model species, males are the pregnant sex, incurring costly investment into the development of brooding structure (Wilson and Orr 2011; Nikolas 2010). Conversely, females produce energy‐rich eggs. *H. erectus* 's unique reproductive biology allowed us to disentangle over sexual maturation, the cost of investment into the brooding structure versus investment into egg production, two costly traits that are commonly associated with female reproduction across many taxa. Seahorses further lack a stomach, leading to unknown responses to food deprivation, as they typically eat at frequent intervals (Woods 2002).

The CR treatment induced a slower growth trajectory (Figure 1A) likely through energy allocation trade‐offs, which aligns with previous research, particularly when implemented during the juvenile stage (Speakman and Mitchell 2011). Compared to the AL males, the CR males exhibited reduced body mass and size after 5 months (Dunn‐test, adj. *p* < 0.0004, Figure 1 and Figure S1B); this effect was slightly less in female seahorses (Dunn‐test, adj. *p* < 0.02, Figure 1A and Figure S1B). Interestingly, an assessment of the condition index revealed that the CR treatment had a less pronounced impact on the overall condition of males relative to females (Tukey HSD, adj. *p* = 0.09 vs. adj. *p* < 0.002, Figure 1B respectively). The results suggest that males are more sensitive to CR in terms of somatic growth, potentially prioritising body maintenance over reproduction. This is reflected in the delayed pouch formation observed in CR males, which occurred approximately 20 days later than in AL males. By day 157, CR males also had a smaller relative pouch size compared to AL males (ANOVA, *p* < 0.03; Figure 1D), suggesting a slower developmental trajectory. This delay may also be attributed to the fact that pouch development is highly resource‐dependent, being a costly process that can only be completed when resources are available, as it is crucial for the individual's fitness.

In contrast, females under CR maintained ovarian weight (ANOVA, *p* = 0.3; Figure 1C) despite experiencing a decline in overall body condition. This suggests a trade‐off between somatic maintenance and reproductive investment, with females prioritising ovarian function even under CR. Since egg production is energetically demanding, CR females may have mobilised body reserves to sustain ovarian tissue, even at the expense of body condition. Additionally, ovarian maintenance may be less energy‐intensive than brood pouch development, allowing females to preserve reproductive potential with minimal energetic input. However, it remains possible that CR negatively affected egg quality or maturation, which may not be reflected in total ovarian weight. The small sample size in the AL group (*n* = 5) limits our ability to detect subtle differences.

These findings suggest a sex‐specific response to CR: males delay reproduction, investing in body maintenance and postponing pouch development, while females sustain reproductive investment at the cost of body condition. This aligns with the disposable soma theory, where males may defer reproduction until conditions improve, avoiding the high energetic costs of developing a vascularised pouch. Interestingly, these results contrast with patterns observed in mammals, where early life adversity often accelerates reproductive development in males. Such differences may reflect distinct life‐history strategies, with seahorse males prioritising somatic growth to develop a larger pouch, which could support a greater number of viable offspring per clutch. Delaying pouch development to later in life may thus enhance overall fitness for males, while females allocate resources to maintaining reproductive potential despite energetic constraints (Isaac 2005).

The cost of nutritional egg provisioning is highlighted by the stark contrast in gene expression between males and females in both gonadal and liver tissue, despite the absence of a significant difference in female ovarian weight between CR and AL (Figure 3). Fasting did not affect sperm production (no DEGs between AL and CR treatments in testes), consistent with findings in conventional model organisms (Kane et al. 2018), likely due to the lower production cost of sperm compared to resource‐intensive egg formation. In the ovaries, 356 DEGs were downregulated (opposed to 10 upregulated) upon CR (Figure 3), with pathways relating to lipid metabolism and the innate immune system (Table S2, sheet C). Also in the liver, female seahorses had more DEGs compared to males (Figure 3) involving similar enriched pathways pertaining to the metabolism of steroids (Table S2, sheet A); however, in the liver, DEGs were predominantly upregulated in CR seahorses. This underscores the reliance of female reproductive functions on energy reserves in adipose tissue, where fat cells produce the hormone leptin, a process diminished during fasting with the loss of body fat (Speakman and Mitchell 2011). Decreased leptin levels correlate with lower progesterone concentrations (Pérez‐Pérez et al. 2015), supported by downregulated *pgr* and *cyp17A2* in the liver of fasting females, which could impair oocyte maturation. Nonetheless, as mentioned earlier, females may prioritise reproduction by investing in egg production even at the cost of egg quality, as indicated by the downregulation of immune responses and lipid genes in the ovaries. This could be a strategy to produce more eggs of lower quality, rather than delaying egg production and potentially missing optimal reproductive windows due to environmental constraints.

Regardless of male seahorses showcasing only a few DEGs upon CR, the liver displayed enriched pathways and genes important for aging, including genes involved in cellular respiration and oxidative phosphorylation (Table S2, sheet B) (Sinclair and Howitz 2005; Speakman and Mitchell 2011). In the liver of CR males, genes controlling circadian rhythm (*cklockb* and *cry3a*), impacted by daily nutrient level fluctuations (Longo and Panda 2016; Manoogian et al. 2022), were upregulated. Studies in mice found sex‐specific signalling of circadian clock genes, with *cry1* being higher in the liver of CR males (Astafev et al. 2017). A well‐regulated circadian clock improves overall health, and this may be particularly important for males delaying reproduction if relying on a longer lifespan to maximise fitness ensures they have the necessary resources for future reproductive efforts.

The downregulation of numerous inflammatory genes (including TLRs 21 and 22, chemokines, tnf, irf5 and M17, known for their pro‐inflammatory roles in fish (Hanington and Belosevic 2007; Xia et al. 2012; Mokhtar et al. 2023)) in CR female ovaries suggests a potential limitation in their capacity to prime their eggs with immunological components for transgenerational plasticity (Beemelmanns and Roth 2016; Roth et al. 2012). This aligns with prior research in the syngnathid *Syngnathus typhle* (Goehlich et al. 2021; Roth and Landis 2017) where exposure to environmental stressors, like elevated temperatures, erased the effects of transgenerational immune priming, potentially driven by a resource allocation trade‐off between survival and immune priming in times of limited resources.

We observed potential beneficial effects of fasting by upregulated genes for increased autophagy (*atg13*) and ketosis (*aacs*) in livers of CR seahorses, in line with other studies (Speakman and Mitchell 2011). The upregulation of both *sesn2* and *tti2* in AL compared to CR (ovarian tissue) results in an activation of mTORC1 and cell growth (Wolfson et al. 2016; Brown and Gromeier 2017). Suppressing mTOR activity may promote longevity by shifting the body from a state of growth to autophagy, thus improving cell homeostasis (Sinclair and Howitz 2005; Speakman and Mitchell 2011). Additionally, *foxO1a* was upregulated in AL ovaries (Figure 3); *FoxO* is typically found elevated in response to high levels of reactive oxygen species (ROS) having a dual role in combating oxidative stress, being both pro‐survival and pro‐apoptotic (Klotz et al. 2015). *FoxO1a* can have positive effects by promoting DNA repair (Klotz et al. 2015). A potential rejuvenation effect in the ovaries of CR females was revealed by higher lipid stores and increased lipid synthesis in AL female ovaries, promoting cell growth compared to fasted females that prioritised pathways for repair and regeneration, shifting from high reproductive output to increased somatic maintenance (Tables S1 and S2, sheet D). Despite the absence of notable morphological effects of CR in females, lipid metabolic pathways were upregulated in the liver and downregulated in the ovaries.

The gut microbiome is crucial for various aspects of health, including vitamin synthesis, immune system fine‐tuning, maintaining the integrity of the intestinal barrier, and the modification of bile acids, neurotransmitters and hormones (Zheng et al. 2018; Doms et al. 2018). Fasting induces changes in mucus production, which in turn affect gut microbial diversity (Angoorani et al. 2021). The gut microbiota play a pivotal role in influencing fish health by fostering the development of the gut epithelium, enhancing the immune system's functioning and acting as a protective barrier, impeding the colonisation of harmful pathogens within the gut (Roeselers et al. 2011; Li et al. 2019). In the CR treatment, *Vibrio* dominated the microbial community, comprising approximately 90% of the abundance in females and 85% in males, significantly higher than in the AL treatment (60% in females and 75% in males) (Figure 4A). This suggests that CR conditions favour *Vibrio* dominance, potentially by creating a more competitive environment where *Vibrio* outcompetes other taxa under resource‐limited conditions. *Vibrio* species can have both beneficial and pathogenic effects, depending on environmental factors and host interactions, which may influence the overall health and microbiome composition of the seahorses (Baker‐Austin et al. 2018).

When *Vibrio* was excluded from the analysis, the CR group showed a more diverse microbial background, with several Proteobacteria genera such as *Pseudoalteromonas*, *Neptunomonas*, *Litorilituus* and others emerging as prominent taxa (Figure 4B). CR seahorses were the strongest driver of variation in the beta‐diversity analysis, with CR individuals being significantly distinct from all other groups (adj. *p* < 0.002, Figure 4D), further emphasising that CR profoundly reshapes the microbial community. The heightened relative abundance and taxonomic diversity within the *Proteobacteria* phylum (Figure S4B) aligns with previous studies suggesting an increased relative abundance of *Proteobacteria* in fasting vertebrates, including fish (Li et al. 2019; McCue et al. 2017). *Proteobacteria*, recognised for their crucial role in preparing the gut for colonisation by strict anaerobic bacteria, play a vital part in maintaining gut health (Shin et al. 2015). In contrast, the AL group exhibited a more balanced microbial community, where the genus *Brevinema* (phylum *Spirochaetota*) emerged as the dominant non‐*Vibrio* genus (Figure 4B).

*Brevinema* relative abundance was closely associated with the expression of several immune genes in the testes (e.g., irf10, mhc1zca, cd74; Figure S5). This association seems to be particularly relevant in the AL treatment, as *Brevinema* was severely reduced in the gut of CR individuals compared to AL (Figure 4B). *Brevinema* is not only a key player in microbial communities associated with fish mucus (Li et al. 2015) and gut microbiota (Lyons et al. 2017), but is also identified as a microbe that is involved in paternal‐specific vertical microbial transfer (Beemelmanns et al. 2019). It is tempting to develop the hypothesis that the CR reduced relative abundance of *Brevinema* connected to a lowered expression of several immune genes results in reduced trans‐generational microbial and immune transfer also on the paternal side under stressful environmental conditions (Roth and Landis 2017). While testing this hypothesis will require investigations on organ‐specific microbiomes, we can currently state more generally that *Brevinema* may play a critical role in immune function, with CR potentially disrupting this relationship and leading to diminished immune responses.

Although beta diversity analysis revealed no significant differences between sexes and treatments (Figure S4D), indicating that the major driver of microbial community differences is the dietary treatment, significant differences in alpha diversity were observed. Specifically, alpha diversity was higher in CR individuals, with a notable difference between CR and AL males (Dunn test, *p* = 0.04), but not females, suggesting that fasting influenced microbial evenness in a sex‐dependent manner. Most types of fasting were shown to increase α‐diversity (Moatt et al. 2016), and our findings align with this background pattern.

On the genus level, *Pseudoalteromonas* exhibited the highest relative abundance (approximately 25% in both males and females) compared to AL, comprising marine species with diverse activities including anti‐bacterial, algicidal, anti‐viral, and specific isolates hindering the settlement of common fouling organisms (Holmström and Kjelleberg 1999). This could confer significant benefits to the immunological health of fasted seahorses. Among the other prevalent taxa in CR, many are commonly found in marine organisms, but their correlation with specific metabolic roles in animals remains unexplored. However, we did find *Neptunomonas*, which has potential nitrate reduction properties (Liu et al. 2013), and *Thalassotalea*, characterised as an aerobic, chemo‐organotrophic genus of bacteria involved in nutrient cycling and the decomposition of organic matter (Zhang et al. 2014). The efficient utilisation of various forms of energy from the environment by these microbes may contribute to their abundance, providing energy for the host during fasting. Additionally, a study revealed that *Litorilituus sediminis* was associated with tumour suppressive activity (Wang et al. 2013), which could also confer some protective properties to CR seahorses. In summary, while the gut in CR conditions may exhibit potential for improved health compared to AL, a clear rejuvenation was not evident. The dominance of certain taxa in CO and CY, consistent across age groups with some variation between sexes, prompts us to consider that a younger gut does not necessarily equate to a healthier state in seahorses.

In conclusion, our findings shed light on the intricate sex‐specific responses to fasting in seahorses, providing valuable insights into the adaptive strategies employed by males and females to cope with nutritional stress and maintain reproductive success. Our study emphasises how the primary determinants of resource allocation are closely tied to sex‐specific life‐history strategies. In *H. erectus* males, this is exemplified by the unique focus on male pregnancy and the allocation of resources post‐copulation, contrasting with the pre‐copulatory investment in female eggs, leading to distinct morphological and regulatory effects of fasting between males and females that are opposed to effects in typical model organisms. Nonetheless, conserved mechanisms of CR were evident in both sexes, but to different degrees, with increased autophagy and ketosis pathways in CR females and enrichment of pathways and genes important for cellular respiration and circadian rhythm control in CR males. As we move forward, additional research in a diverse range of model species with varying reproductive strategies and sex roles will be essential to build upon our findings. Understanding the intricate connections between reproduction, resource availability and fasting effects can contribute to a more comprehensive understanding of sex‐specific health, lifespan dimorphism and evolutionary adaptations in response to nutrient scarcity, particularly in the context of climate change. In an ecological context, these sex‐specific fasting responses could have important implications for species survival, particularly in environments where nutrient availability fluctuates, influencing reproductive timing and success and shaping evolutionary strategies in the face of climate change. By adopting a multidisciplinary approach, researchers can make significant strides in translating the findings into practical applications for health and beyond.

## Author Contributions

F.A.P. and O.R. conceived and designed the study. F.A.P. conducted the experiment, analysed the data, interpreted the results, and wrote the manuscript. C.F.E. extracted RNA and DNA. V.A.W. analysed the 16S rRNA sequencing data. O.R. supervised the project and contributed to manuscript writing. All authors reviewed and approved the final version of the manuscript.

## Ethics Statement

The work was carried out in accordance with the German animal welfare law and with the ethical approval given by the Schleswig‐Holstein Ministerium für Energiewende, Landwirtschaft, Umwelt, Natur und Digitalisierung (MELUND) (permit no. 1315/2021). No wild endangered species were used in this investigation.

## Conflicts of Interest

The authors declare no conflicts of interest.

## Supporting information


**Figure S1.** Morphological and physiological differences across dietary treatments and age.
**Figure S2.** Principal Component Analysis (PCA) plot illustrating the separation of organs and treatment groups based on the first two principal components.
**Figure S3.** Bar plot illustrating sex‐biased gene expression in seahorses under ad libitum (AL) and caloric restriction (CR) dietary treatments.
**Figure S4.** Microbial diversity and composition across treatment and age groups in seahorses.
**Figure S5.** Multidimensional Scaling (MDS) analysis of microbial composition and gene expression in male *Hippocampus erectus* testes tissue across dietary treatments.
**Table S1.** Quantitative results from the differential gene expression (DGE) analysis (using *limma:voom*) comparing different groups.

**Table S2.** Results from gene enrichment analysis using g:Profiler for *Hippocampus erectus* comparing caloric restriction (CR) and ad libitum (AL) feeding groups. Panel A: AL females versus CR females for liver gene enrichment, Panel B: AL males versus CR males for liver gene enrichment, Panel C: AL females versus CR females for ovary gene enrichment, Panel D: CR females versus control old (CO) females for ovary gene enrichment. Gene enrichment includes *Danio rerio* orthologues, and pathways assessed include Gene Ontology (GO) terms, KEGG pathways and Reactome pathways.

**Table S3.** Differential gene expression analysis for the caloric restriction (CR) experiment in *Hippocampus erectus* . Results include five contrast comparisons: (A) ad libitum (AL) females versus CR females, (B) AL males versus CR males, (C) all AL individuals versus all CR individuals, (D) AL females versus CR females (duplicate contrast) and (E) CR females versus control old (CO) females.

**Table S4.** Results of 16S rRNA amplicon sequencing analysis in *Hippocampus erectus* , showing microbial composition across experimental groups.

**Table S5.** Morphological raw data for *Hippocampus erectus* in the caloric restriction (CR) experiment. Panels include (A) weight (g) and length (cm) of individuals under CR and ad libitum feeding, (B) weight and length of young (4 months) and old (≥ 3 years) individuals, and (C) weight and length of individuals euthanised before the end of the CR experiment.

**Table S6.** Statistical analyses for *Hippocampus erectus* across different datasets. Panels include (A) statistical analysis of morphological measurements by dietary treatment; (B) statistical analysis of gene expression count data from liver, head kidney and gonads; (C) statistical analysis of 16S rRNA amplicon sequencing from gut tissue; and (D) gradient analysis between bacterial taxa (gut tissue) and transcriptomic variation (liver, head kidney and gonads).

## Data Availability

Additional information is available in the Supporting Information. Refer to Tables [Link], [Link], [Link], [Link], [Link], [Link] for comprehensive details: Table S1 presents an overview of numerical outcomes derived from the *limma* DGE analysis, encompassing various groups and organs. For the results of the GO analysis, consult Table S2. Visual insights from the DGE analysis concerning the specified organs and treatment groups are presented in Figure S3. Table S4 offers the results of the 16S rRNA amplicon sequencing analysis conducted in QIIME2, detailing all OTUs at the genus level. Table S5 encompasses morphological raw data, specifically the length and weight measurements of all CR, AL, CY and CO seahorses. Table S6 displays the full statistical analysis results for morphology, RNA‐Seq and 16S rRNA‐Seq. The raw sequencing RNA‐Seq data, 16S rRNA‐Seq and metadata used in this study are available from the National Center for Biotechnology Information (NCBI) Sequence Read Archive (SRA) under BioProject ID PRJNA1004169 (RNA‐Seq: SUB13725814; 16S rRNA‐Seq: SUB13759493).
